# 21-Gene assay as predictor of chemotherapy benefit in HER2-negative breast cancer

**DOI:** 10.1038/s41523-018-0090-6

**Published:** 2018-11-14

**Authors:** Charles E. Geyer, Gong Tang, Eleftherios P. Mamounas, Priya Rastogi, Soonmyung Paik, Steven Shak, Frederick L. Baehner, Michael Crager, D. Lawrence Wickerham, Joseph P. Costantino, Norman Wolmark

**Affiliations:** 1 NRG Oncology/NSABP (NSABP Legacy trials are now part of the NRG Oncology portfolio), Pittsburgh, PA USA; 2 Massey Cancer Center, Virginia Commonwealth University, Richmond, VA USA; 3 The University of Pittsburgh, Pittsburgh, PA USA; 4 Orlando Health UF Health Cancer Center, Orlando, FL USA; 5 The University of Pittsburgh Cancer Institute, Pittsburgh, PA USA; 6 Yonsei University College of Medicine, Seoul, Republic of Korea; 7 Genomic Health, Inc., Redwood, CA USA; 8 Allegheny Health Network Cancer Institute, Pittsburgh, PA USA

## Abstract

The NSABP B-20 prospective-retrospective study of the 21-gene Oncotype DX Breast Cancer Recurrence Score® test predicted benefit from addition of chemotherapy to tamoxifen in node-negative, estrogen-receptor positive breast cancer when recurrence score (RS) was ≥31. HER2 is a component of the RS algorithm with a positive coefficient and contributes to higher RS values. Accrual to B-20 occurred prior to routine testing for HER2, so questions have arisen regarding assay performance if HER2-positive patients were identified and excluded. We report an exploratory reanalysis of the B-20, 21-gene study following exclusion of such patients. Patients were considered HER2 positive if quantitative RT-PCR for HER2 was ≥11.5 units, and excluded from re-analyses performed using the original cutoffs: <18, 18–30, ≥31, and the TAILORx cutoffs: <11, 11–25, >25. The endpoint remained distant recurrence-free interval (DRFI) as in the original study. Distribution was estimated via the Kaplan–Meier method and compared via log-rank test. Multivariate Cox proportional hazards models estimated chemotherapy benefit in each group. In the RS < 18 and 18–30 groups, 1.7 and 6.7% were HER2 positive. In the RS ≥ 31 group, 41% were HER2 positive. Exclusion resulted in fewer events, with loss of significance for benefit from chemotherapy in the overall HER2-negative cohort (log-rank *P* = 0.06), but substantial benefit from chemotherapy remained in the RS ≥ 31 cohort (HR = 0.18; 95% CI: 0.07–0.47) and the RS > 25 cohort (HR = 0.28; 95% CI: 0.12–0.64). No benefit from chemotherapy was evident in the other RS groups. Following exclusion of HER2-positive patients based on RT-PCR expression, substantial benefit of chemotherapy remained for RS ≥ 31 as originally employed, and with RS > 25 employed in TAILORx.

## Genetics: multigene panel predicts chemo benefit regardless of HER2 status

A commonly used genetic test helps to predict whether patients with estrogen-receptor positive, node-negative breast cancer stand to gain from chemotherapy. Charles Geyer from NRG Oncology/NSABP and Virginia Commonwealth University and colleagues reanalyzed data from a decades-old study that helped establish a 21-gene panel as a tool for determining whether to add chemotherapy to endocrine therapy for women with estrogen-receptor positive, node-negative breast cancer. The researchers excluded patients with HER2-positive disease because *HER2* gene expression is part of the diagnostic test and questions have persisted regarding the performance of the assay, if HER2-positive patients were excluded. This new analysis demonstrated that although chemotherapy did not extend disease-free survival overall among the cohort of patients with HER2-negative disease, it still proved beneficial for those with high recurrence scores on the genetic test. The findings thus validate the clinical utility of the test for patients with estrogen-receptor positive, HER2-negative, node negative breast cancers.

## Introduction

The prospective-retrospective NSABP B-20 trial evaluating the Oncotype DX® 21-gene assay as a predictor of benefit from adjuvant chemotherapy in node-negative, estrogen-receptor (ER)-positive breast cancer demonstrated that a high recurrence score (RS), defined as 31 or higher, was predictive of chemotherapy benefit.^1,2^ In preparation for the TAILORx trial, the analysis of the B-20 trial was repeated with the cutoffs ultimately employed in TAILORx: <11, 11–25, >25, and demonstrated that the patients with RS > 25 also had a large benefit from the addition of chemotherapy.^3^ Supportive findings of chemotherapy benefit in patients with RS ≥ 31 were subsequently demonstrated in a similar prospective-retrospective analysis of SWOG-8814, conducted in patients with node-positive, ER-positive breast cancer.^4^

The 21-gene assay is based on RT-PCR analysis and integration of expression of 16 breast cancer-related genes and 5 reference genes.^5^ HER2 is one of the genes in the RS algorithm with a positive coefficient and contributes to a higher RS value. As a result, cancers with HER2 overexpression generally have higher RS values. In NSABP B-20, patients were accrued from October 1988 to March 1993, prior to establishment of routine clinical testing for HER2, so a portion of the patients accrued to B-20 were likely to have had HER2-positive disease.^6^

Formal tests for interaction between quantitative individual HER2 gene expression as well as expression of the HER2 gene group by RT-PCR and benefit from chemotherapy were conducted as part of the original analysis of the 21-gene assay and were negative, so positive HER2 status should not be a prerequisite for benefit from chemotherapy in patients with high RS.^5^ However, because the 21-gene assay is used clinically as a predictive biomarker of chemotherapy benefit for patients with hormone-receptor positive, HER2-negative disease, questions have persisted regarding the performance of the 21-gene assay in B-20 if patients with HER2-positive tumors were excluded. Insufficient tumor material remains in the blocks used in the prospective-retrospective B-20 study to allow sectioning for routine immunohistochemical (IHC) or in situ hybridization (ISH) testing for HER2 expression without risk of wastage of the remaining material. However, the quantitative HER2 individual gene score component of the RS assay was compared to assessment of HER2 status by FISH in a case–controlled study using specimens from patients identified in the Kaiser Permanente Northern California Cancer Registry. The study demonstrated concordance of 97% (95% CI: 96–99%) by central FISH and the RS assay with a definition of HER2 positive as quantitative RT-PCR ≥ 11.5 units.^7^ A second study^8^ using 901 specimens from Alliance N9831 compared HER2 status determined by RT-PCR using the same cutoff with results determined by IHC and FISH. Concordance for HER2 assessment by RT-PCR was 95% vs. IHC and 91 % vs. FISH. Although quantitative RT-PCR has not been validated as a companion diagnostic for identifying individual candidates for HER2-directed therapies, the concordance with HER2 determination by IHC and FISH documented in these two large studies justifies use of the single-gene expression by RT-PCR to identify the patients in B-20 who were likely to have been HER2-positive by routine testing methods.

We report here for the first time results of an exploratory reanalysis of chemotherapy benefit in B-20 when excluding the cohort of patients with quantitative RT-PCR ≥ 11.5 units, in order to demonstrate the performance of the assay in predicting chemotherapy benefit for patients with node-negative, ER-positive, HER2-negative disease. In addition, we assessed the performance of the 21-gene assay in predicting chemotherapy benefit in the B-20, HER2-negative population using the RS cutoffs employed in the TAILORx study.^3^

## Results

### Characteristics of the study population

**Fig. 1:**
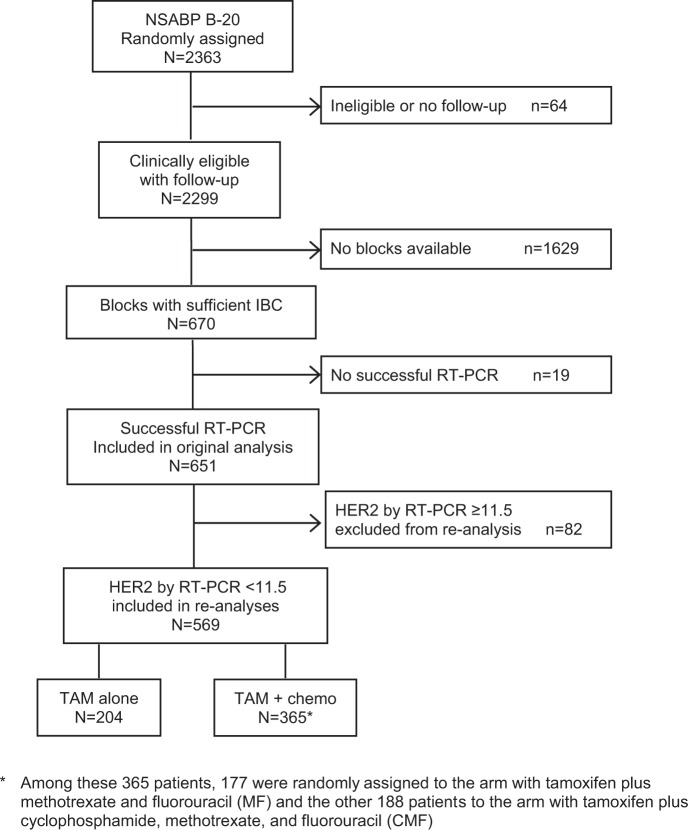
CONSORT Diagram for 21-gene assay study using available primary breast cancer specimens from NSABP B-20

Based on a quantitative RT-PCR cutoff of ≥11.5 units to define HER2-positive disease, we identified and excluded 82 patients (12.6%) from the B-20 cohort used for the original 21-gene study as shown in Fig. 1. Among the patients with RS < 18 and RS 18–30, only 6/353 (1.7%) and 9/134 (6.7%), respectively, had quantitative RT-PCR for HER2 ≥ 11.5 and were excluded. In contrast, among patients with RS ≥ 31, 67/164 (40.9%) had quantitative RT-PCR for HER2 ≥ 11.5 and were excluded from these analyses.

The characteristics of the study population after exclusion of patients identified with HER2-positive disease defined by quantitative RT-PCR ≥ 11.5 are shown in Table 1. Patient age, tumor grade, tumor size, and receptor status were generally similar between treatment groups, except for grade as determined by Pathologist B.

**Table 1:** Summary of covariates, excluding HER2-positive disease

	TAM alone (*n* = 204)	Chemo + TAM (*n* = 365)	All (*n* = 569)
*Patient age, y*			
Median (minimum–maximum)	51 (31–74)	51 (28–74)	51 (28–74)
*Tumor grade* *(central Pathologist A assessment)*			
Well differentiated	56 (27.7%)	92 (25.2%)	148 (26.1%)
Moderately differentiated	86 (42.6%)	187 (51.2%)	273 (48.1%)
Poorly differentiated	60 (29.7%)	86 (23.6%)	146 (25.7%)
Missing	2	0	2
*Tumor grade* *(central Pathologist B assessment)*			
Well differentiated	28 (13.8%)	86 (23.6%)	114 (20.1%)
Moderately differentiated	132 (65.0%)	176 (48.2%)	308 (54.3%)
Poorly differentiated	43 (21.2%)	103 (28.2%)	146 (25.7%)
Missing	1	0	1
*Tumor grade (site assessment)*			
1	21 (11.8%)	52 (15.8%)	73 (14.4%)
2	107 (60.5%)	191 (57.9%)	298 (58.8%)
3	49 (27.7%)	87 (26.4%)	136 (26.8%)
Missing	27	35	62
*Tumor size*			
≤1.0 cm	30 (14.9%)	67 (18.4%)	97 (17.1%)
1.1–2.0 cm	106 (52.8%)	174 (47.8%)	280 (49.5%)
2.1–4.0 cm	58 (28.8%)	112 (30.8%)	170 (30.0%)
≥4.1 cm	8 (4.0%)	11 (3.0%)	19 (3.4%)
Unknown	2	1	3
ER			
0–9 fmol/mg	0	0	0
10–49 fmol/mg	78 (38.2%)	137 (37.5%)	215 (37.8%)
50–99 fmol/mg	53 (26.0%)	92 (25.2%)	145 (25.5%)
100–199 fmol/mg	36 (17.7%)	72 (19.7%)	108 (19.0%)
200+ fmol/mg	37 (18.2%)	64 (17.5%)	101 (17.8%)
PR			
0–9 fmol/mg	21 (10.3%)	61 (16.7%)	82 (14.4%)
10–49 fmol/mg	29 (14.2%)	63 (17.3%)	92 (16.2%)
50–99 fmol/mg	28 (13.7%)	55 (15.1%)	83 (14.6%)
100–199 fmol/mg	39 (19.1%)	62 (17.0%)	101 (17.8%)
200+ fmol/mg	87 (42.7%)	124 (34.0%)	211 (37.1%)

### Analyses based on original RS cutoffs of 18 and 31

**Fig. 2:**
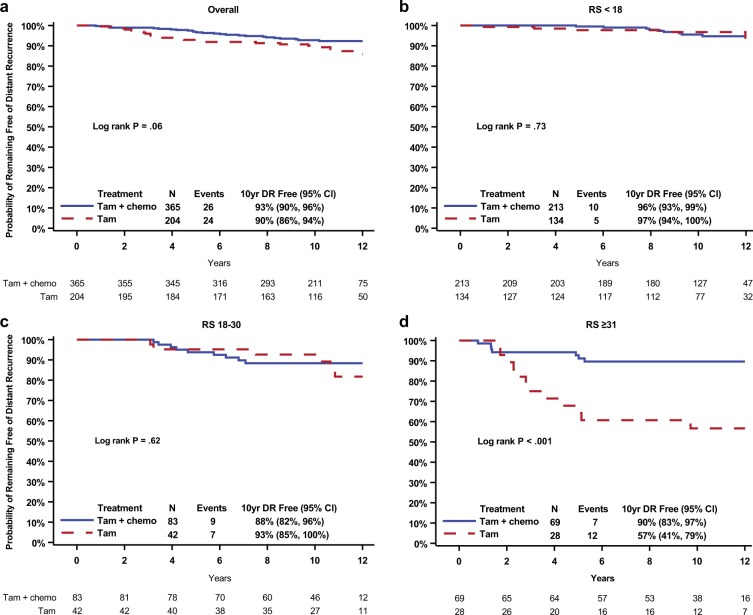
Kaplan–Meier estimates of the probability of remaining free of distant recurrence by original RS groups following exclusion of patients with presumed HER2-positive disease, comparing treatment with tamoxifen alone with tamoxifen plus chemotherapy. **a** All patients; **b** low-risk RS < 18; **c** intermediate-risk RS 18–30; and **d** high-risk RS ≥ 31

Kaplan–Meier plots were used to assess the evidence of prediction for chemotherapy benefit for the overall study population and were categorized by the original RS cutoffs, <18, 18–30, and ≥31, as shown in Fig. 2. Although the exclusion of patients with HER2-positive disease resulted in a reduction in events, with an associated loss of statistical significance for benefit from the addition of chemotherapy to tamoxifen in the overall B-20 population (log-rank *P* = 0.06, Fig. 2a), there remained a highly significant benefit from chemotherapy in the RS group with RS ≥ 31 (HR = 0.18; 95% CI: 0.07–0.47; *P* < 0.001, Fig. 2d) with Kaplan–Meier estimates of the proportion of patients distant recurrence-free at 10 years at 56.7% (95% CI: 47.2–66.2) in the patients treated with tamoxifen compared to 89.6% (85.9–93.3%) with the addition of chemotherapy. There was no evidence of benefit from chemotherapy in the RS < 18 and RS 18–30 groups (Fig. 2b, c).

The test for interaction between chemotherapy treatment and RS was statistically significant (*P* = 0.023) in the multivariable model that simultaneously adjusted for patient age, tumor size, ER, PR, and tumor, as shown in Table 2.

**Table 2:** Cox Proportional Hazards Regression Model^a^ assessing interaction of recurrence score (RS) risk group (Grouping Method I) with chemotherapy effect, excluding patients with HER2-positive disease

*n* = 564 patients		
Effect	Hazard ratio (95% Confidence interval)	Likelihood ratio test on interaction, *P* value
Chemotherapy in RS < 18	1.19 (0.40–3.49)	0.023
Chemotherapy in RS from 18–30	0.64 (0.23–1.75)	
Chemotherapy in RS ≥ 31	0.18 (0.07–0.46)	

^a^ Adjusting for patient age (>50 vs. ≤50 years), clinical tumor size (>2.0 vs. ≤2.0 cm), ER by ligand binding assay (≥100 vs. <100 fmol/mg), PR by ligand binding assay (≥100 vs. <100 fmol/mg), and tumor grade (well differentiated, moderately differentiated, and poorly differentiated). Five (5) patients had missing values in tumor grade or tumor size.

### Analyses based on TAILORx RS cutoffs of 11 and 25

**Fig. 3:**
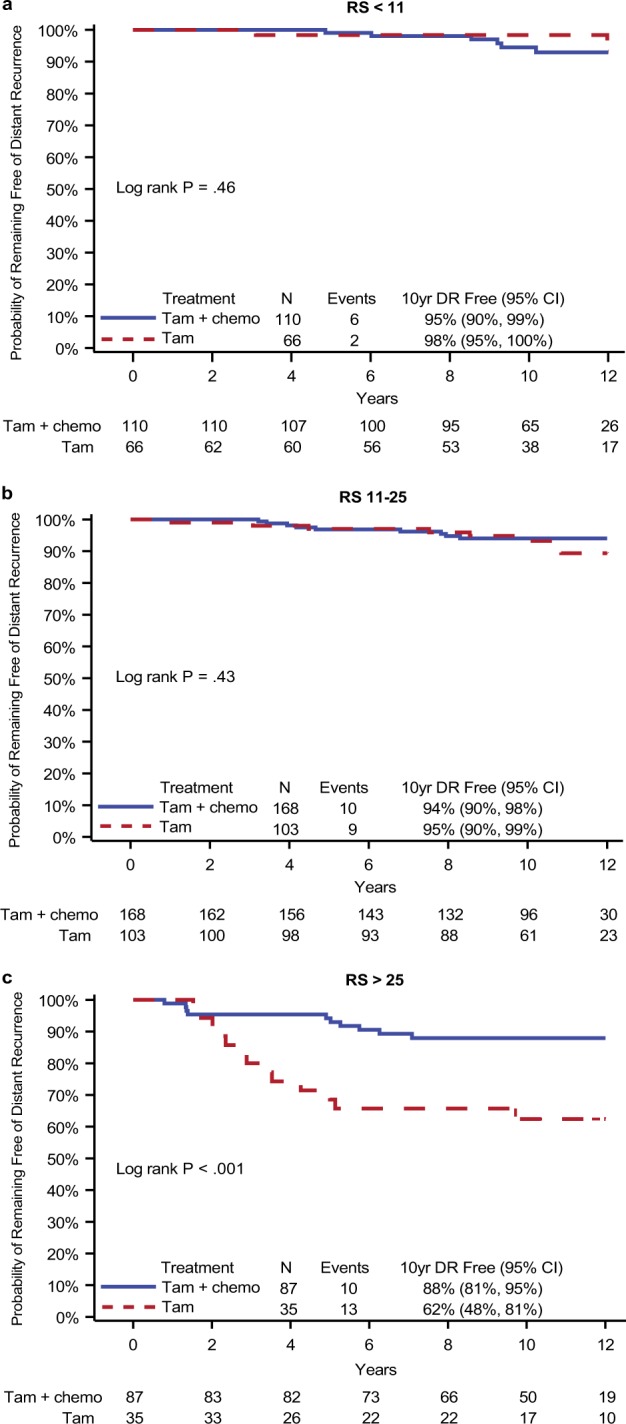
Kaplan–Meier estimates of the probability of remaining free of distant recurrence by TAILORx RS groups following exclusion of patients with presumed HER2-positive disease comparing treatment with tamoxifen alone with tamoxifen plus chemotherapy. **a** RS < 11; **b** RS 11–25; **c** RS > 25

Kaplan–Meier plots were also used to assess the evidence of prediction for chemotherapy benefit categorized by the TAILORx cutoff criteria, <11, 11–25, >25, as shown in Fig. 3. A statistically significant benefit from the addition of chemotherapy to tamoxifen was present in the high RS > 25 group (HR = 0.27; 95% CI: 0.12–0.62; *P* < 0.001, Fig. 3c) with a 10-year distant recurrence-free estimate at 62% (95% CI: 48%–81%) in the patients treated with tamoxifen compared to 88% (81%–95%) with the addition of chemotherapy (absolute distant recurrence risk reduction 25.5%, 95% CI: 7.8%–43.2%). Among the women ≤50 years the HR for improvement with addition of chemotherapy was 0.12 (95% CI: 0.03%–0.49%) and among the women >50 years the HR was 0.44 (95% CI: 0.14%–1.37%). There was no evidence of benefit from chemotherapy in the RS < 11 and RS 11–25 groups (Figs. 3a, b). The test for interaction between chemotherapy treatment and RS groups was statistically significant (*P* = 0.014) in multivariable models that simultaneously adjusted for patient age, tumor size, ER, PR, and tumor grade, as shown in Table 3.

**Table 3:** Cox Proportional Hazards Regression Model^a^ assessing interaction of recurrence score (RS) risk group (Grouping Method I) with chemotherapy effect, excluding patients with presumed HER2-positive disease

*n* = 564 patients		
Effect	Hazard ratio (95% Confidence interval)	Likelihood ratio test on interaction, *P* value
Chemotherapy in RS ≤ 10	1.19 (0.41–3.51)	0.014
Chemotherapy in RS from 11–25	0.61 (0.26–1.35)	
Chemotherapy in RS > 25	0.27 (0.12–0.62)	

^a^ Adjusting for patient age (>50 vs. ≤50 years), clinical tumor size (>2.0 vs. ≤2.0 cm), ER by ligand binding assay (≥100 vs. <100 fmol/mg), PR by ligand binding assay (≥100 vs. <100 fmol/mg), and tumor grade (well differentiated, moderately differentiated, and poorly differentiated). Five (5) patients had missing values in tumor grade or tumor size.

## Discussion

Following publication of the results of the original analysis of the 21-gene assay as a predictive biomarker for chemotherapy benefit in NSABP B-20,^1^ expert panels recommended the assay for clinical use to determine potential benefit from the addition of chemotherapy to endocrine therapy in patients with ER-positive, HER2-negative, node-negative breast cancer.^9,10^ Because the clinical utility of the assay as a predictor of chemotherapy benefit has been limited to patients with hormone-receptor positive, HER2-negative disease, questions have persisted regarding the performance of the 21-gene assay in the B-20 patient population if the patients with HER2-positive tumors were excluded, even though interaction testing between HER2 expression and chemotherapy benefit in the original study was negative.^1^ We report here results of an exploratory reanalysis of the original B-20 data from 21-gene assay following exclusion of patients with HER2-gene expression units ≥ 11.5, who were likely to have been HER2 positive as assessed by conventional IHC and ISH methods.

Relatively few patients were identified as HER2 positive in the RS < 18 and RS 18–30 groups, but a substantial percentage of patients in the RS ≥ 31 group (41%) were identified as HER2 positive. Following exclusion of all patients identified as having presumed HER2-positive disease, reanalysis demonstrated a large benefit of chemotherapy remained for patients whose breast cancers were positive for ER, presumably negative for HER2, and who had an RS ≥ 31. These findings reinforce the findings of the original interaction testing, which provided no evidence that the inclusion of patients with undocumented HER2-positive disease accounted for the benefit from chemotherapy reported in the original publication.

Exclusion of the presumed HER2-positive patients for this study reduced the overall study population from 651 to 569 patients and resulted in loss of statistically significant evidence (log-rank *P* = 0.06) for the chemotherapy benefit in the overall HER2-negative study population. However, the estimated HR of 0.59 (95% CI: 0.31–1.04) was consistent with the original 21-gene study (HR = 0.56, 95% CI: 0.34–0.91, log-rank *P* = 0.02), as well as the parent NSABP B-20 study (HR = 0.63, 95% CI: 0.49–0.81, log-rank *P* < 0.001), indicating that the loss of significance was related to the reduced sample size.

In planning TAILORx, a large prospective study of the 21-gene assay in ER-positive, HER2-negative, node-negative early breast cancer, the trial Steering Committee adjusted the RS cutoffs from 18 and 31 to 11 and 25 to minimize the potential for under treatment in both the high-risk group and the randomized group.^3^ As previously published, patients in the RS < 11 group treated with endocrine therapy alone had an excellent outcome.^11^ Patients in the RS 11–25 group were randomized to endocrine therapy alone or endocrine therapy plus chemotherapy and results of this primary analysis were recently published.^12^ All patients with RS > 25 were to be treated with chemotherapy and endocrine therapy, with choice of therapy at investigator discretion, and then followed. Because the results of TAILORx will become integrated into clinical practice, we elected to also re-analyze the prospective-retrospective B-20 study cohorts following exclusion of the patients with presumed HER2-positive disease using the cutoffs of 11 and 25 from TAILORx. Following exclusion of patients with presumed HER2-positive breast cancer, substantial benefit from the addition of chemotherapy to tamoxifen in patients with RS > 25, was clearly demonstrated. These results provide justification for use of this cutoff to identify an important minority of patients with ER-positive, HER2 negative, node-negative early breast cancer who are at high risk for distant recurrences without chemotherapy, and who should be offered chemotherapy to substantially reduce the risk of distant recurrences.

## Methods

### Patients

Gene expression results of a 21-gene assay were obtained in 651 patients, with 227 tamoxifen-treated and 424 chemotherapy-treated, from the NSABP B-20 study previously reported.^1^ Details on the sample preparation, list of reference and cancer-related genes, and the computing algorithm were presented in Paik et al.^5^ The study was approved by the Essex Institutional Review Board (IRB; Lebanon, NJ), the Allegheny General Hospital IRB (Pittsburgh, PA), and the University of Pittsburgh IRB (Pittsburgh, PA). All study participants consented in the NSABP B-20 study and the need for additional informed consent for the substudy on the 21-gene assay was waived by the IRBs. All analyses in this study were based on data from the NSABP B-20 patients identified as HER2 negative based on the HER2-gene expression (<11.5) from the RT-PCR assay of the 21-gene assay.

### Endpoint

The endpoint was distant recurrence-free interval, defined as time from random assignment to first distant recurrence. Local, regional recurrence, and second primary cancers were ignored. Patients who died of causes other than cancer were censored at death.

### Statistical analysis

The Kaplan–Meier method was used to estimate the proportion of patients free of distant recurrence over time and the log-rank test with a two-sided *P* value was used to compare Kaplan–Meier curves.^13^ Multivariate Cox proportional hazards models were used to examine the interaction between chemotherapy treatment and RS as a continuous variable, adjusting for patient age (>50 vs. ≤50 years), clinical tumor size (>2.0 vs. ≤2.0 cm), ER by ligand binding assay (≥100 vs. <100 fmol/mg), PR by ligand binding assay (≥100 vs. <100 fmol/mg), and tumor grade (well, moderate, and poor).^14^ The likelihood ratio test for interaction compared the reduced model, which excluded the RS by treatment interaction, with the competing full model, which included the RS by treatment interaction. A one-sided *P* value < 0.05 for the likelihood ratio test was considered significant. Multivariate Cox proportional hazards models were also performed using two different methods for defining RS-risk categories: <18, 18–30, and ≥31; 0–10, 11–25, and >25, to estimate the chemotherapy benefit within each RS-risk group. Equality of chemotherapy benefit, expressed as a hazard ratio, across RS-risk groups defined by each of the two grouping methods was tested using a likelihood ratio test. A one-sided *P* value < 0.05 was considered statistically significant.

### Code availability

All analyses were performed with the statistical packages SAS/STAT 9.4 or R (Version 3.4).^15,16^ The SAS program followed standard SAS codes for using SAS Proc Lifetest and Proc Phreg.^15^ The R program used standard R code for the R package “Survival.”^16^

## Data Availability

The clinical data were from NSABP (now part of NRG Oncology), and the assay data from Genomic Health, Inc., were merged by an honest broker and may be obtained upon agreement from both NRG Oncology and Genomic Health, Inc.
